# Left behind: newcomer children with disabilities and their families

**DOI:** 10.3389/fpubh.2024.1332403

**Published:** 2024-01-23

**Authors:** Sukaina Dada, Nida Khan, Naila Dewji

**Affiliations:** SMILE Canada Support Services, Mississauga, ON, Canada

**Keywords:** newcomer, refugee, intersectionality, disability, pediatric, immigrant, social support

## Introduction

December 3rd is a day when governments and private institutions worldwide commemorate the *International Day of Persons with Disabilities*. Proclaimed by the United Nations, this day aligns with the promise of the 2030 Agenda for Sustainable Development to *leave no one behind and uphold the rights of all disabled persons* (1). However, disabled newcomers in Canada, and parents of children with disabilities, which include refugees and newly settled immigrants, are continually left behind and repeatedly excluded from disability-related policies and disability-specific services (2).

Upon settling in a new country, newcomers have difficulty accessing adequate education, meaningful employment, financial independence, housing and food security, rehabilitation services, and social support (3). These challenges are further exacerbated for newcomer children with disabilities and their families, as they cannot effectively navigate healthcare and social services due to language, cultural, and financial barriers (3). As a result, they are missing out on essential funding opportunities and access to critical services, including therapies, respite services, and social programs.

SMILE Canada—Support Services, a charity formed in 2008 to address the barriers that newcomer children with disabilities and their families face in Canada, specifically from underserved and underrepresented Muslim communities, models how culturally responsive support essential for families can be implemented in programming and service delivery. Over the years, SMILE has increasingly seen registration of families from all around the world, including Somalia, Syria, Palestine, Sudan, and Afghanistan. SMILE Canada's critical work highlights one example of how organizations can bridge gaps in services and support by advocating for the intersectional needs of children with disabilities and their families and providing critical programs, including culturally responsive service navigation, language-specific parent support groups, and social and educational programs.

Today, in the current geo-political and socio-economic context, when xenophobia and Islamophobia are on the rise in Canada (4), newcomer children with disabilities and their families require safer, culturally responsive resources and support *now more than ever before*.

## A priority population

By 2036, the population of newcomer residents in Canada is estimated to increase from ~24.5 to 30% (5). These include families who are displaced due to war, poverty, and climate change. They leave behind their homes, families, careers, and established support networks and must learn to traverse Canada's complex healthcare systems (3). Canada has prioritized the resettlement of vulnerable families, which includes disabled persons, and half of those settled are under the age of 15 (6).

Newcomer parents face challenges accessing needed support for their children due to significant out-of-pocket expenses of healthcare services, communication and language barriers, and transportation limitations (3). Additionally, research findings suggest that disabled newcomers face challenges related to stigma, barriers in accessing health information and an absence of culturally appropriate care (7). These challenges lead to delays in seeking and receiving acute and outpatient treatment, which have detrimental impacts (7). In hospital settings, research has shown that mistrust between patients and clinicians further jeopardizes patient care, resulting in fewer families accessing health care when needed (8). SMILE families often report hesitancy and fear of seeking help from healthcare and education providers, as their lived experiences are discounted, and their identities and oppressions are overlooked and overpowered. For these families, there are few inclusive services and available opportunities, a lack of awareness of available services and supports, and language differences that can ultimately lead to communication barriers between clients and providers (9).

## The need for culturally responsive care

The ongoing narratives of exclusion of disabled newcomer children and their families drive staff at SMILE to go into communities locally and across the country and challenge service providers to re-evaluate their inclusive practices. Understanding narratives that highlight the intersectional needs and oppressions of disabled newcomers will inform changes in policies and practices that contribute to their marginalization. Authentic narratives and research can expose policies and practices that discriminate against disabled newcomers and their families, limiting them from navigating healthcare and education systems and accessing resources and supports.

Parents and caregivers at SMILE report that they require unique wellness support tailored to their challenges and experiences, which includes intergenerational trauma. Families are subjected to financial barriers and exclusionary practices in their daily lives and communities, leading to feelings of isolation, exclusion, lack of belonging, and overall depleted mental health. Although there are existing social services, they are not specific to the racialized and diverse communities that SMILE works with, lack cultural understanding and culturally safer approaches, and are unaffordable and inaccessible.

Research has shown that differences in health outcomes, also known as the social determinants of health, for newcomers compared to Canadian-born residents result from cultural and language differences that create challenges in being meaningfully included into Canadian society (10). The social determinants of health are heavily influenced by the availability of culturally appropriate mental health and quality of life services within communities (11). Coupled with more than three in five Canadians with disabilities experiencing at least one communication barrier, either in understanding or being understood (12), current models of service delivery have yet to adopt holistic culturally responsive approaches to reduce barriers to access and limit adverse health outcomes.

Studies have shown that information limitations of European American-based service provision systems, limited access to opportunities, stereotyping, and communication/language difficulties are just some of the many obstacles that racialized individuals with disabilities, especially those who identify as Muslim, face when trying to seek mainstream services (9). These barriers stem from a lack of culturally responsive care, highlighting an identified need as this lack impacts how families understand and cope with a diagnosis, alongside the treatment plans they undertake. For example, despite the diagnosis, a newcomer's difficulty in communicating and understanding diagnoses can further be exacerbated when coupled with stereotyping by service providers, eventually leading to ineffective treatment plans, failed follow-through on treatment, and limited follow-up with clinicians. Research has shown the positive effects culturally responsive care can have on long-term treatment for newcomers with disabilities. In cases with children with Autism Spectrum Disorders (ASD) specifically, culturally responsive professionals had more productive sessions with immigrant families (13). Instead of the traditional monolingual communication for families with ASD, Yu (13) found it would be advantageous to offer linguistically suitable bilingual language services to immigrant families. Additionally, a shared background helped educators understand the child and increased the parent's access to services (13).

Considering the needs of newcomers with disabilities when creating solutions is vital to building an equitable society where everyone, regardless of race, age, culture, faith, and ability, can engage and actively participate in daily living. Current models of service delivery fail to consider the diverse needs of newcomers with disabilities, with a lack of holistic and culturally competent care inhibiting these communities from having their needs met (14). Understanding the importance of culturally responsive care is the first step in implementing culturally safe and beneficial practices to yield more positive outcomes for families.

## Discussion

Newcomers with disabilities must be a priority as they face many barriers upon arriving in their host countries. We must strive to provide culturally relevant and responsive services to foster safer spaces, advocate for fundamental human rights, and stand against the numerous oppressions families face. Creating culturally responsive services and having care relevant to families can significantly reduce perceptions and instances of discrimination (8) and lessen marginalization (15).

Culturally responsive care is a nuanced proposition. It includes increasing service providers' knowledge and training and addressing power imbalances and personal biases that are deep-rooted in the Canadian healthcare system. Cultural responsiveness requires policy decision-makers to evaluate their own personal and cultural privileges and examine the role of Canada's colonial history and how it affects organizations at a systemic level (16). Training on cultural safe practices and culturally responsive care to service providers and organizational staff is imperative in building more equitable communities. Training and awareness on adopting an equity framework when implementing policies and practices can help build stronger relationships and trust with populations with intersecting needs. This includes adopting anti-oppressive, anti-racism and anti-black racism, anti-ableist, anti-Islamophobic, and trauma-informed approaches. Incorporating these aspects of culturally responsive support can create an environment for those seeking care to feel respected and safe.

As we celebrate the International Day of Persons with Disability, we must be self-critical and ask ourselves, “Who are we leaving behind?” The refugee and newcomer disabled community in Canada is often left behind in policy and decision-making, service delivery and provision, and receiving adequate accommodations and support. Exploring our biases and privileges and tackling systemic discrimination within our institutions is required to support disabled newcomers in Canada. Doing so will have more successful outcomes, such as families accessing services they need and desire, not services forced upon them. A one-size-fits-all approach only benefits one population; traditionally, it is a white middle-class population.

It is time for us to rewrite policies so that they address multiple intersectional forms of oppression and educate healthcare professionals, service providers, and educators on the significant forms of oppression that impact disabled refugees. Intentional awareness of transnational disablement and xenophobic ableism (17) and the need to identify and listen to stories of disabled newcomers will impact service provision in healthcare and education.

Diversity, Equity and Inclusion work continually excludes dis/ability and ableism from conversations on inclusion. It rarely involves intersectional experiences and oppression (18), including newcomers, specifically refugees with disabilities, a population caught between a national and transnational narrative. While conversations on diversity, equity and inclusion can be viewed as a step forward in various disciplines, disabled newcomers must be included within that fold if these topics are to be addressed with sincerity and a firm commitment to culturally sensitive policies.

## Author contributions

NK: Writing – original draft, Writing – review & editing. SD: Writing – original draft, Writing – review & editing. ND: Writing – original draft, Writing – review & editing.
